# The Tessellated Monkey: Parsing the Functional Fields of the Auditory Cortex

**DOI:** 10.1371/journal.pbio.0040245

**Published:** 2006-06-20

**Authors:** Liza Gross

No self-respecting concertgoer of a certain era would consider wearing earplugs at a show, but that was long before Pete Townsend and other rock icons spoke out about the risk of deafness. Today, most people recognize that high-intensity noise causes hearing loss—except maybe for those iPod users who routinely blast earsplitting music straight into their brains.

Blaring volume causes deafness by destroying sound-responsive hair cells, but it's unclear how these auditory assaults affect the brain's auditory system. Much of the auditory cortex is organized by sound frequency, but neuroscientists are still figuring out the extent of the spatial organization of frequency-selective neurons and how each auditory field contributes to sound perception. While neurophysiological studies have characterized the functional properties of certain auditory cortical fields (by recording the electrical activity from individual neurons), anatomical studies have identified other fields that had not been functionally characterized.

A new study by Christopher Petkov, Nikos Logothetis, and colleagues fills in some of these gaps by using high-resolution functional magnetic resonance imaging (fMRI) on macaque monkeys presented with acoustic stimuli. The researchers used the anatomical and neurophysiological data to see how the fMRI data compared with the already described auditory cortical fields. With a better sense of how to interpret the functional imaging data, they could use fMRI to probe the functional properties of uncharacterized auditory fields. This approach allowed them to show the functional organization of 11 discrete auditory fields in the primate auditory cortex—an important step toward understanding how these fields operate together to shape what the primate listener perceives of its acoustical environment.

Petkov et al. first used a broad spectrum of sound frequencies to globally activate the monkeys' auditory cortex. (Six anesthetized monkeys and one monkey trained to stay still were placed in fMRI scanners while presented with acoustical stimuli.) Next, they used low- and high-frequency sounds to identify regions with selectively tuned neurons. Based on predictions that auditory fields follow an alternating pattern of high to low frequency along a posterior to anterior direction, they expected fMRI activity to follow the same pattern—which it did. This now “grounded” frequency gradient allowed them to match the rest of the activity patterns that they observed with other auditory fields. Significantly, they matched an alternating pattern of high- and low-frequency selective regions with three fields in the primary auditory cortex, or auditory “core” fields: A1, R, and RT. These core fields are thought to be surrounded by seven or eight so-called “belt” (non-primary) fields. However, neurophysiological data on RT and many of the belt fields are scant, making it unclear how many functional fields actually exist.

It's thought that auditory fields in the core are tuned to simple sounds, like single-frequency tones, while fields in the belt respond to complex sounds. To better locate activity in the belt regions, Petkov et al. also studied brain responses to more-complex sounds. The results included frequency selectivity patterns consistent with known patterns for four belt fields that had previously been studied neurophysiologically and provided a base outline for the other fields—basically functionally tessellating the monkey auditory cortex. Petkov et al. then took advantage of evidence that tones produce a stronger response in the core than they do in the belt fields to outline a border between the core and belt, which helped them to further resolve the position of the core relative to the belt fields.

The extensive patterns of frequency gradients indicated that the three core regions were surrounded by eight belt fields, four on each side, supporting anatomical evidence for about a dozen auditory fields. The researchers then went on to show that neurons in the belt fields responded preferentially to sounds with a broad frequency spectrum—in other words, more complex sounds that would have some of the properties of natural sounds. These results fall in line with a model of hierarchical auditory processing in which the core operates during the initial stages of auditory cortex processing, contributing to a frequency analysis of the sounds in the environment. The belt fields function at a higher level to deal with more-complex sounds by integrating sound frequencies. The challenge now is to understand how each of the many fields contributes toward and interacts with others to shape the perception of primates in their typically opulent acoustical—and multisensory—environments.

This study underscores the value of pooling data from different experimental approaches to study something as intricate as the brain. With this high-resolution functional MRI map of the monkey auditory cortex, researchers can now use both fMRI and neurophysiological techniques to refine each field's particular role within the primate auditory cortex. The map will also guide efforts to better understand the functional organization of the human auditory system—information that could certainly identify the impact of peripheral hearing loss on this part of the auditory system. And with functional maps of both monkey and human auditory cortex, researchers can better understand how the specialized auditory fields evolved, ultimately offering insights into the evolution of human language.

## 

**Figure:**
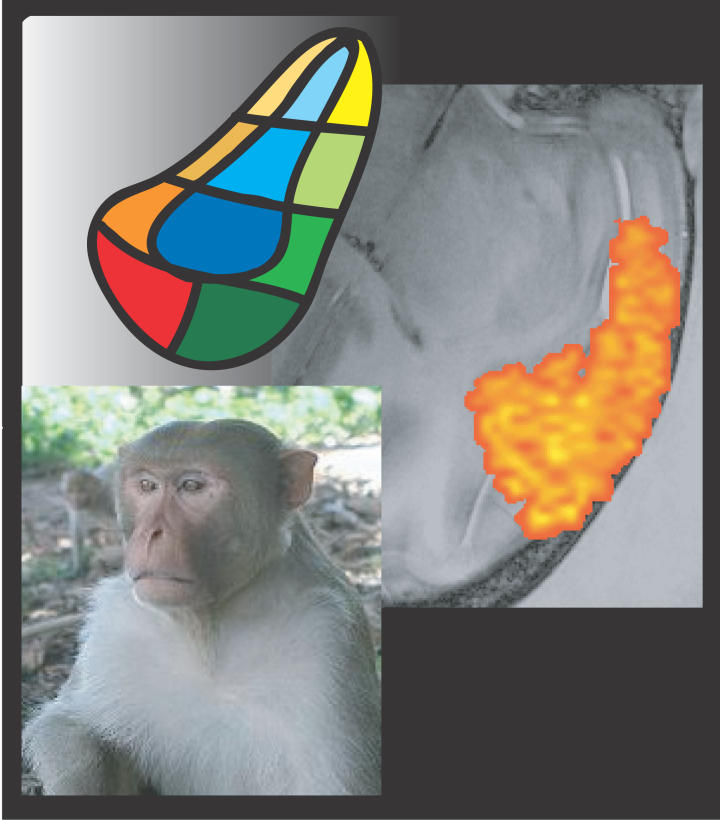
Non-invasive functional imaging reveals many discrete functional areas in the auditory cortex of a non-human primate. (Photo: Marc D. Hauser)

